# Non-Destructive Evaluation of Rock Bolt Grouting Quality by Analysis of Its Natural Frequencies

**DOI:** 10.3390/ma13020282

**Published:** 2020-01-08

**Authors:** Mario Bačić, Meho Saša Kovačević, Danijela Jurić Kaćunić

**Affiliations:** Faculty of Civil Engineering, University of Zagreb, 10000 Zagreb, Croatia; msk@grad.hr (M.S.K.); djk@grad.hr (D.J.K.)

**Keywords:** rock bolt, grouting quality, dynamic response, natural frequency, finite element method

## Abstract

Grouted rock bolts represent one of the most used elements for rock mass stabilization and reinforcement and the grouting quality has a crucial role in the load transfer mechanism. At the same time, the grouting quality as well as the grouting procedures are the least controlled in practice. This paper deals with the non-destructive investigation of grouting percentage through an analysis of the rock bolt’s natural frequencies after applying an artificial longitudinal impulse to its head by using a soft-steel hammer as a generator. A series of laboratory models, with different positions and percentages of the grouted section, simulating grouting defects, were tested. A comprehensive statistical analysis was conducted and a high correlation between the grouting percentage and the first three natural frequencies of rock bolt models has been established. After validation of FEM numerical models based on experimentally obtained values, a further analysis includes consideration of grout stiffness variation and its impact on natural frequencies of rock bolt.

## 1. Introduction

**Figure 1:**
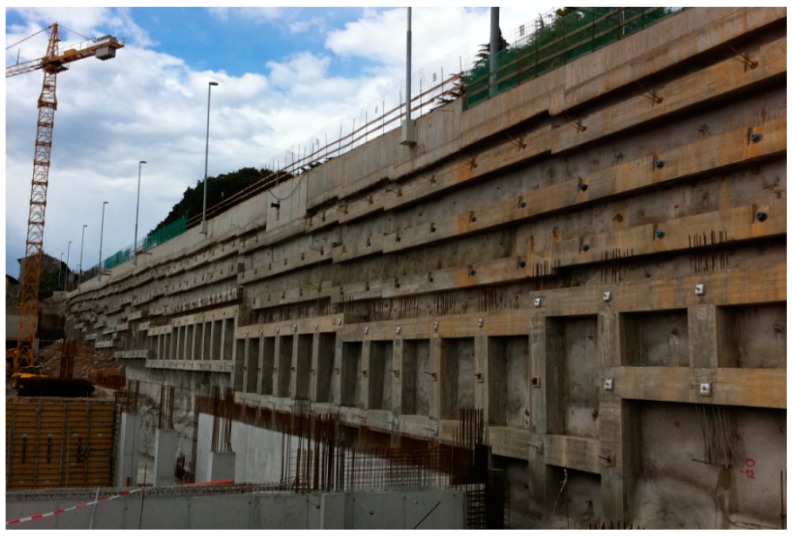
An example of the installed rock bolt system.

Ensuring the stability of rock mass excavation is a challenging task for all persons involved in a construction process. Any kind of instability, which is a result of an inadequate support system can lead to undesirable temporal, financial and, what is most important, safety consequences. For decades, there has been a practice of using cement-based grouted rock bolts for rock mass reinforcement and much experience, both positive and negative, has been gained through their use. Rock bolts are an inevitable part of a large number of projects, such as rock slopes (Figure 1), where different levels of rock bolt design are used, ranging from empirical, analytical, and numerical, the latter having the most expanding applications.

**Figure 2:**
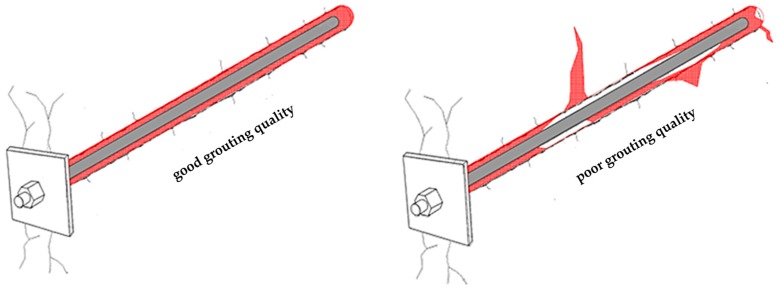
Good and poor grouting quality of a rock bolt.

Regardless of the design method used and the level of detail of the rock bolt parameter assignment, the usual practice is to take into consideration fully grouted rock bolts, without any grout defects along their length. Although some authors [1] have stated that grouted rock bolts present the optimal reinforcement method for strengthening weak or fractured rocks, a number of authors point to the fact that such rock masses may cause issues with grouting quality. For example, application of grouted rock bolts in karstic rock mass, characterized by the presence of voids and discontinuities, can lead to the severe grout loss [2,3], limiting their applicability in these types of rock masses [4,5]. The presence of karstic phenomena can lead to poor grouting, as shown in Figure 2. However, most of rock mass discontinuities are non-persistent, even though their visible traces can lead to conclusions as if they were open fractures. Assumption of full persistence gives incorrect predictions of hydraulic conductivity, as well as of grout flow. Since the visible trace length of a discontinuity can be a poor indicator of true persistence, some innovative methods, such as FERM given by Shang et al. [6] may help to detect the internal connections of rock bridges that hinder the flow of grout.

Although the grouted rock bolts can produce a higher degree of load transfer in comparison to the other types of bolts, the grouting procedures and characteristics of the bonds between the bar and the grout and with the rock mass are the least controlled in practice [7], while the rock mass grouting procedures remain a predominantly empirical practice [8]. The importance of grouting quality can be seen through the potential rock bolt failure mechanisms, where most significant mechanisms include pull-out failures at bar–grout (B–G) contact, as well on grout–rock mass contact (G–RM). The main role of the grout is to provide a mechanism for transferring the load between the rock mass and the reinforcement element, and grouting quality has an important role in this task, along with the interface strength between bolt, grout, and rock, influenced by the adhesion, friction, and mechanical interlocking. Ren et al. [9] conclude that, based on many laboratory and in situ tests, the most common failure type of fully grouted rock bolts is at the bar–grout or grout–rock mass interface. It can be understood that if the grout and rock have similar strengths and if the required grouted rock bolt length is inadequate, then failure could occur at the bolt–grout interface [10]. If the surrounding rock mass is softer, as is the often case with karst, then the failure could happen at the grout–rock interface. There are other pull-out mechanisms which are not directly linked with grouting quality but depend on rock mass characteristics. However, these are out of scope of this paper. The relevant equations for the rock bolt pull-out capacities are:

(1) $$R_{B\text{-}G}\;=\;d_{BR}\cdot \pi \cdot l\cdot \mu \cdot \tau_{B\text{-}G}$$

(2) $$R_{G\text{-}RM}\;=\;d_{BH}\cdot \pi \cdot l\cdot \mu \cdot \tau_{G\text{-}RM}$$

The product of bar diameter *d_BR_* (m) or borehole diameter *d_BH_* (m), installed rock bolt length l (m), π and unit shear strength of bar–grout contact *τ_B-G_* or unit shear strength of grout–rock mass contact *τ_G-RM_* (kN/m^2^), theoretically give the values of pull-out capacity at bar–grout contact *R_B-G_* (kN) and pull-out capacity at grout–rock mass contact *R_G-RM_* (kN), respectively. Since the pull-out resistance can be achieved only on the grouted portion of a rock bolt, it is of importance to properly determine the actual grouting percentage of the installed rock bolt, µ (—). It is a common designer’s assumption of a fully grouted rock bolt (µ = 1), but due to the above-mentioned reasons, a µ < 1 value is more appropriate for the rock mass characterized by the presence of voids and discontinuities.

Much research has dealt, with greater or lesser success, with problems of determining the rock bolt grouting quality taking in consideration its importance for the pull-out capacity. Bačić et al. [11] and Song et al. [12] give the overview of the testing methods. Among many acoustic methods, the first one developed for determining grouting quality is the Boltometer method, which has several limitations when applied to soft and highly fractured rock masses. By using this method, the assumption of a lower sound velocity in the grout in comparison to the velocities in the bar and rock mass is made [13], but this may not be the case with karstic rock masses characterized by low stiffness [14]. If the rock mass has similar stiffness as the grout, a large portion of the energy will dissipate in the rock mass, making the reflections more difficult to recognize [15]. Some progress in this field was made through analysis of ultrasound guided waves [16], where the upgraded version of the Boltometer is currently in development under the name of rock bolt tester [17]. When it comes to vibration-based methods, the GRANIT system [18] stands out and it is getting frequently implemented in practice. This non-destructive system is however dominantly oriented towards determining the force in an anchor. Still, many findings of the GRANIT development process have been significant for the implementation of vibration based non-destructive testing of these types of structural elements. Kovačević et al. [3] had the basic objective of determining the dominant frequency, determined from the power spectrum, of rock bolt models and developing a correlation between the dominant frequency and the grouting percentage. A laboratory testing was conducted on 16 models representing different grouting defects and constructed of 25 mm bar embedded in square concrete blocks of 25 cm side length, having the total length of 3 m. Additionally, 30 field models of 3 m length were tested on location of a tunnel. Overall, the research was not continued and no clear link between the dynamic response and the grouting quality was established, but the authors stress that the procedure is very attractive for further research. Other methods based on an analysis of the frequency response are in development, but need further improvement. The potential of using electrical (such as time-domain reflectometry, electrical resistance, or the Mise-a-la-Masse method) and electromagnetic methods (such as ground penetrating radar) for determining grouting quality have been considered by many authors [19,20,21], but these methods have not found wider application in this domain.

This paper presents a research on the possibility of determining grouting percentage of rock bolts based on an analysis of the dynamical response by considering its natural frequencies after the generation of an artificial impulse to its head. By considering three natural frequencies instead of analyzing only the dominant frequency in the spectrum, as was done by Kovačević et al. [3], a more concrete correlation could be established so that the value of grouting percentage factor (µ) used for verification of designed grouting assumptions is determined in a more reliable manner.

## 2. Theoretical Background

**Figure 3:**
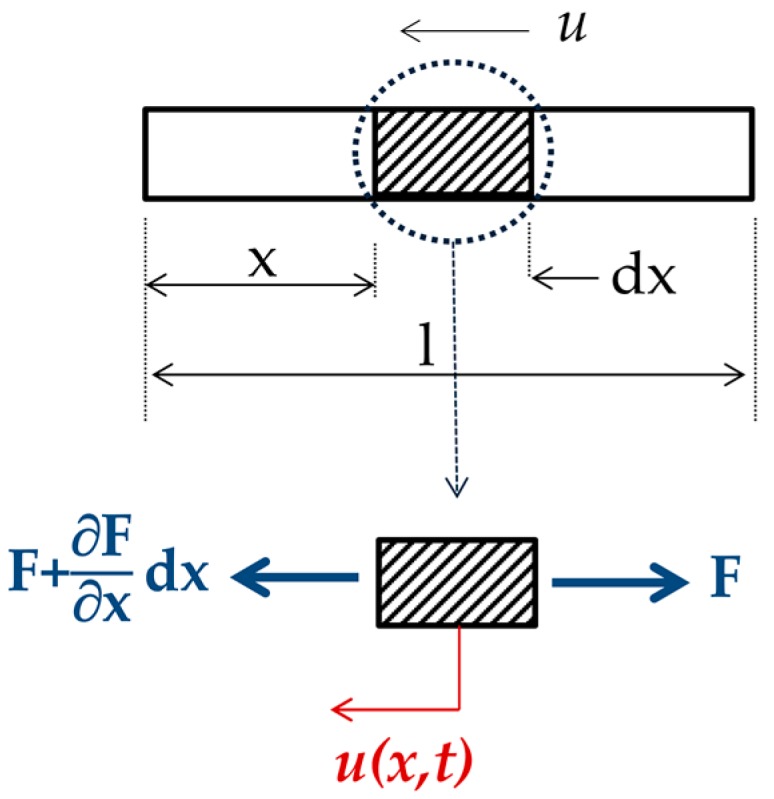
A segment of a bar with position of infinitesimal element.

Vibration based inspection (VBI) is a research domain, which has found its place not just in rock engineering but also in other branches of engineering. Much research has dealt with direct or inverse solutions [22], that is, with the assessment of the effect of structural damage on its parameters as well as with the problem of detecting, locating, and quantifying the extent of the problem. In order to understand the behavior of longitudinal wave propagation in a bar, a certain explanation must be given. Figure 3 shows a segment of a bar with cross section A, material density r, and elasticity modulus E, where the infinitesimal element of δx is in equilibrium. If the bar is loaded with dynamical force F(x,t) in the direction of its axis, it will yield a displacement u(x,t).

By solving the equation:

(3) $$\frac{\partial F}{\partial x}\;=\;\rho A\frac{\partial^{2}u}{\partial t^{2}}$$

the natural frequencies of the bar can be obtained.

By rearrangement of the equation, while employing the expression for the velocity $v\;=\;\sqrt{\frac{E}{\rho }}$ and considering a solution in the form:

(4) u(x,t) = U(x) · (Acosωt + Bsinωt)

where *A* and *B* are constants depending on the initial conditions and *ω* is the angular frequency, Equation (3) becomes:

(5) $$\frac{\partial^{2}U}{\partial x^{2}}\;+\;\frac{\omega^{2}}{v^{2}}U\;=\;0$$

and it has a solution of the form:

(6) $$U\;=\;M\cos\frac{\omega x}{v}\;+\;N\sin\frac{\omega x}{v}$$

where *M* and *N* are constants depending on the bar’s boundary conditions.

In the present paper, as will be shown later, it is of interest to determine the natural frequencies of a rock bolt model, where a system with both ends fixed, as well as a system with one end fixed and the other free, is considered. Due to the infinite number of solutions there are an infinite number of natural frequencies, one for each *n* = 1, 2, 3, …, which can be determined by solving Equation (3):

(7) $$\omega_{n}\;=\;\frac{n\pi }{L}\sqrt{\frac{E}{\rho }}\;(\mathrm{for}\;\mathrm{both}\;\mathrm{fixed}\;\mathrm{ends})$$

(8) $$\omega_{n}\;=\;\frac{\left(2n\;-\;1\right)\pi }{2L}\sqrt{\frac{E}{\rho }}\;(\mathrm{for}\;\mathrm{one}\;\mathrm{end}\;\mathrm{fixed},\;\mathrm{the}\;\mathrm{other}\;\mathrm{free})$$

## 3. Experimental Testing of Rock Bolt Models

### 3.1. Rock Bolt Models

**Figure 4:**
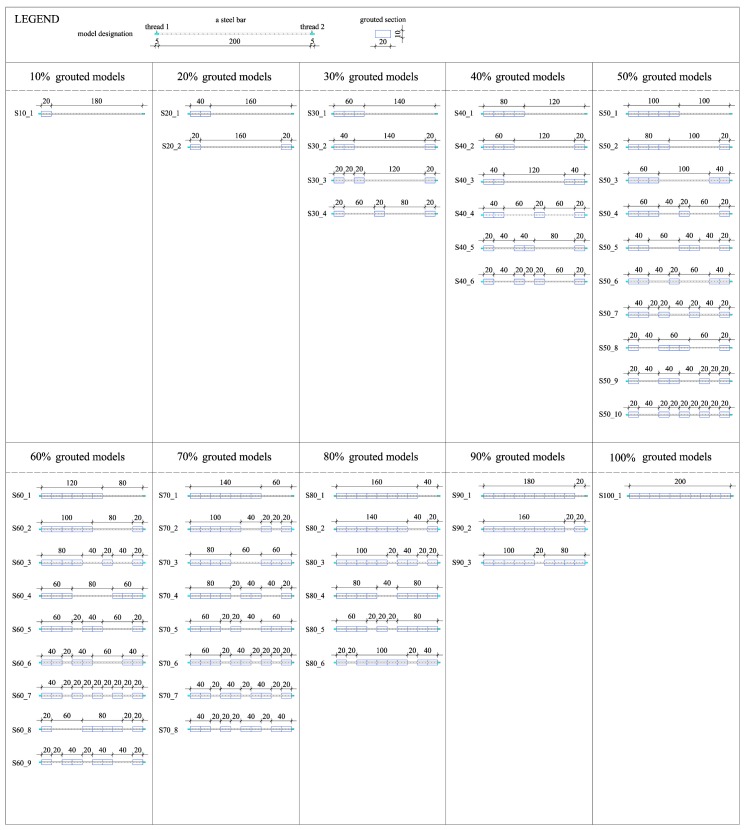
Complete scheme of rock bolt laboratory models.

For the purpose of this research, 51 physical models of grouted bars were made and tested at 1:1 scale. The models were designed to simulate different cases of grouting from the aspect of different grouting percentages and in regard to the position of the grout. Since steel rods of 2100 mm (2000 m bar with 50 mm thread on each side of a bar) were used and the grouting sections had a resolution of 10% of the total length of the rod, the grouted and non-grouted sections were 200 mm long. Therefore, a grouting section is represented with a 200 × 100 × 100 mm element (length × width × height) and the steel bar of 25 mm diameter is centered within this section. Figure 4 shows the schemes of model combinations. The reason for many models is to cover as large as possible a range of grouting percentages and defect positions. Based on the schemes and considering the possibility of generating impulses on both sides of the steel rod (50 mm bar threads are made on each side), a total of 94 testing combinations in respect to different grouting percentages and grout positions were made. In addition, the natural frequencies of the bar alone, i.e., 0% grouted percentage, were determined.

**Figure 5:**
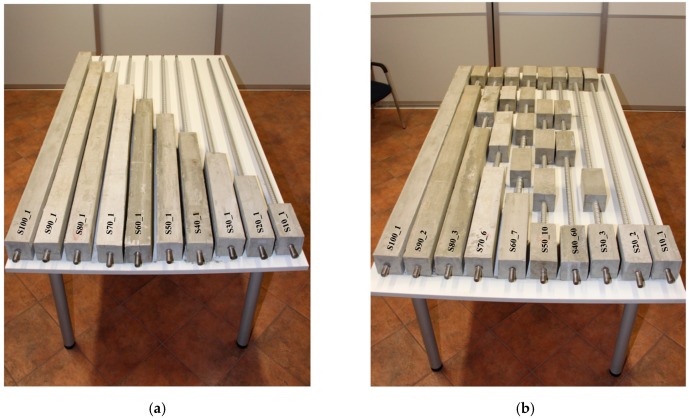
Rock bolt laboratory models: (**a**) Group I and (**b**) Group II.

After the wooden framework was filled with a grout based on the grouting scheme from Figure 4, and after smoothing of the upper surface, the framework was removed, and models were left to reach a 28-day strength before being subjected to the tests. Figure 5a shows, as an example, ten models that have a regular increase in the length of the grouted section. Figure 5b refers to, as an example, ten randomly selected rock bolts from each group of different grouting percentages. These two groups will be later used for numerical modelling.

### 3.2. Characteristics of the Models’ Materials

**Figure 6:**
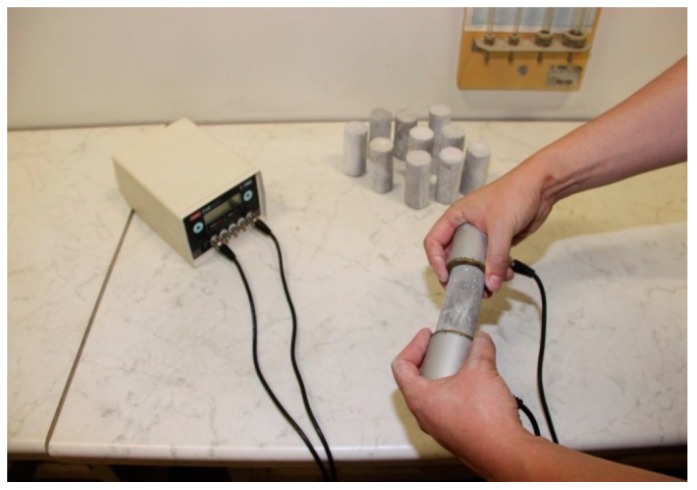
Conduction of ultrasound testing on grout samples.

For the construction of the physical models, the reinforcing steel bars B500B with 25 mm diameter were used. Since the parameters of the steel bar can be considered as ‘reliable’, in the sense that these steel bars were produced under strictly controlled manufacturing conditions, no additional tests for determination of their geometrical and physical and mechanical parameters were performed. In the present study, a cement-based mixture was used as the grout, made from pure Portland cement, water, and filling while no additives were used. During the preparation of the grout, samples were continuously taken in order to determine the physical–mechanical and chemical characteristics. A w/c ratio of 0.42 was used for all models. The laboratory tests to which all samples, were subjected after reaching the 28-day strength are:

1. Determination of the sample length (L), diameter (d), and mass (m) in order to determine its density (r).
2. Ultrasonic test, Figure 6, used to determine the elastic wave propagation velocity through samples. Based on this, the small strain stiffness could be determined for each sample.

**Figure 7:**
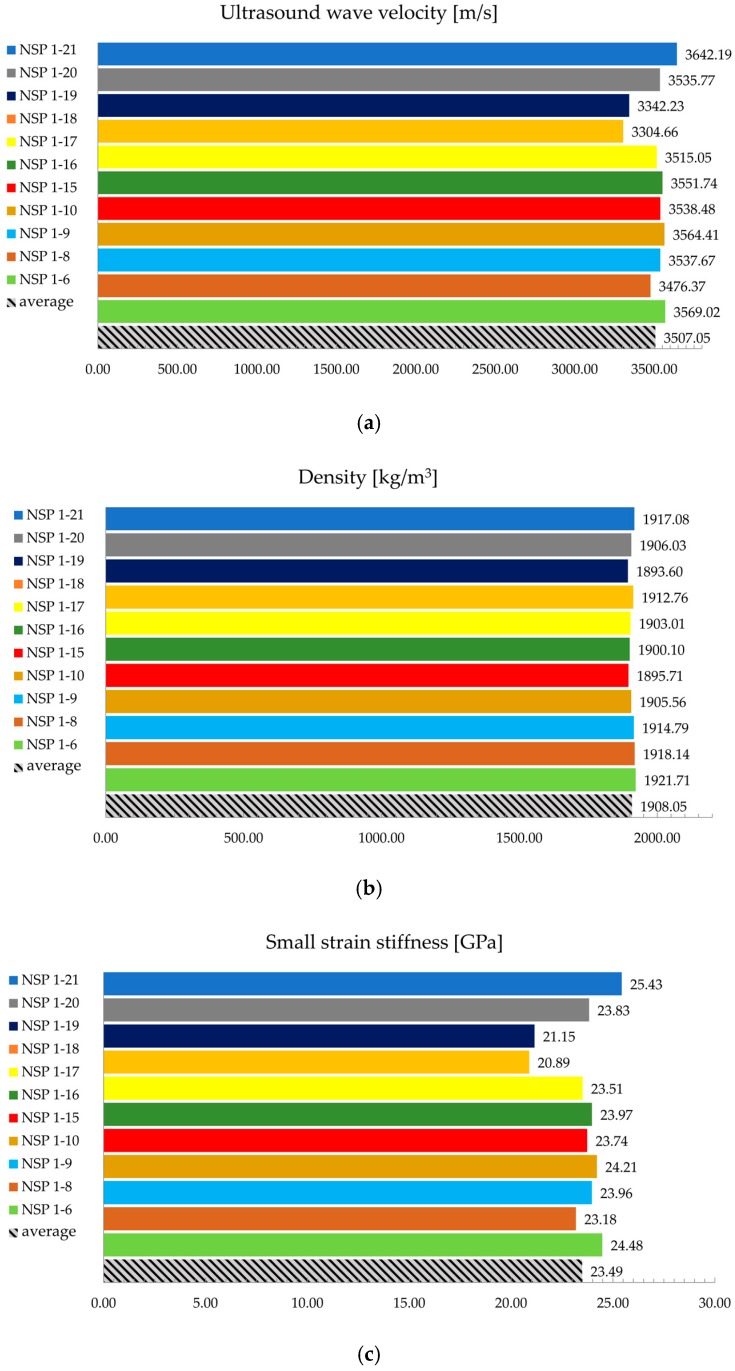
Laboratory test results for 30% grouted model: (**a**) ultrasound wave velocity, (**b**) density, and (**c**) small strain stiffness.

All tests were documented, and the results were used as an input for subsequent numerical simulations. Figure 7 shows a wave velocity determined by the ultrasonic tests (a), density (b), and stiffness at a small strain (c). In particular, the figure shows the results for samples taken for the purpose of preparing a rock bolt model with 30% of grouted section, as an example. Since numerical analyses require only one value input, the testing results are averaged. This procedure has been carried out for samples of all rock bolt models.

It could be seen from Figure 7 that the difference in wave velocities for all samples is up to 10%, while the density difference is up to 1.5%. A small strain stiffness difference is up to 18% which is expected taking into consideration that the square of wave velocity value is implemented into the equation. These exemplary results are in line with laboratory test results for all samples taken for preparation of all rock bolt models.

### 3.3. Acquisition Equipment

**Figure 8:**
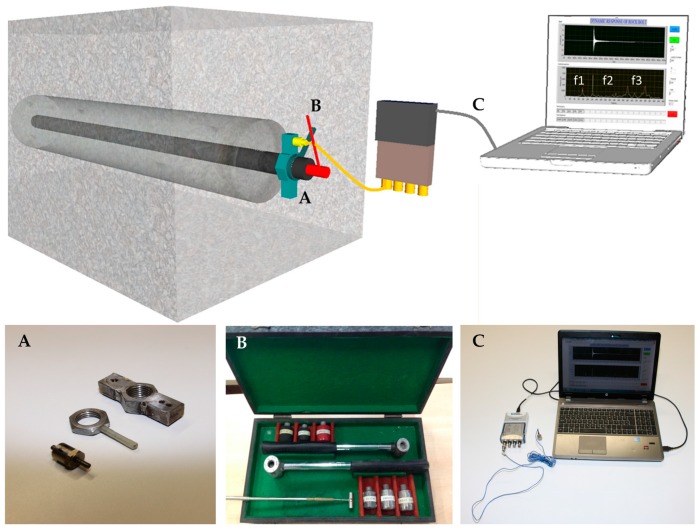
Elements of acquisition equipment: (**A**) a custom made nut with accelerometer; (**B**) a hammer for generation of impulse and (**C**) a laptop with developed state machine and a vibration input module.

The equipment for acquisition of the rock bolt’s dynamical response consisted of several elements, Figure 8, including a custom made nut with accelerometer, a hammer for generation of impulse and a laptop with developed state machine and a vibration input module.

At one end of the model, a nut is fitted on which an accelerometer for detection of longitudinal waves is mounted. The sensitivity of the accelerometer is 10.2 mV/(m/s^2^), with a frequency range of 5 to 15,000 Hz, which has proved to be sufficient for this research. To generate an impulse at the top of the rock bolt, a soft-steel hammer was used. The impulse generated by a hammer is not an ideal delta function but has a certain time duration, which is, along with the shape of the frequency function, determined by the mass and rigidity of the hammer and the steel bar. Therefore, a soft-steel head proved to be the optimal form among an array of materials (hard steel, lead, rubber, wood, plastic). The registered signal goes through a vibration input module, which has built-in anti-aliasing filters that automatically adjust to define the sample rate. After arriving at the developed state machine, the signal is transformed from the time to the frequency domain by using the Fourier transformation.

Considering that even the accelerometer positioning nut itself influences the frequency response, it is necessary to determine such undesirable frequencies in order to eliminate them from the frequency spectrum. Therefore, a calibration system for the direct positioning of the accelerometer on the rock bolt head using a magnet was developed. Even though, in this case, the impact of the positioning equipment is minimized, such positioning cannot be considered as acceptable in real case conditions, because it would be impossible to generate an impact on the rock bolt head. By testing laboratory models, a magnet can be positioned at one end of the bar and the impulse imposed on the other end of the bar. Thus, the frequency response can be compared with the frequency response of the system using the nut as an integral part of the equipment. By overlapping these two spectra, conclusions as to the nut’s ‘contribution’ from the aspect of the additional, undesirable, frequencies in the spectrum can be deduced.

It was found that the nut, after generating an impact on the rock bolt head, vibrates at higher frequencies than those of interest in this research. By overlapping the frequency spectrums, it can be concluded with a high level of reliability that the first three frequencies from the spectrum correlate relatively well, whereas at higher frequencies, especially at those greater than 4000 Hz, there is a significant difference in the frequency response. If only first three natural frequencies are considered, the proposed mode of accelerometer positioning using a nut can be considered acceptable. This was confirmed for all 94 tested combinations.

### 3.4. The State Machine

**Figure 9:**
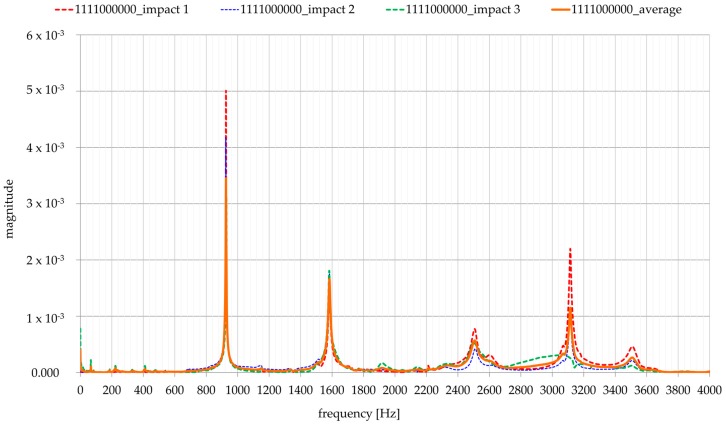
Frequency spectrum averaging procedure.

A state machine, developed within this research, provides the ability to collect data in the time domain and their real-time transformation into the frequency domain, thus gaining insight into the frequency spectrum already during the investigation. As a programming tool, the LabVIEW platform was used, where the concept of so-called ‘state machines’ is implemented. This concept relies on three elements necessary for proper functioning [23]: States, events, and actions. Several states were developed including ‘acquire’ state, ‘analyses’ state which includes implementation of the fast Fourier transform (FFT), ‘save’ state, ‘wait’ state, and ‘stop’ state. All these states provide a faster and more efficient data collection and analysis. After the user has defined all the states, the user interface is visible, where the user has, at any time, access to representations of the signal in the time domain and in the frequency domain. Additionally, a stacking procedure involving averaging three frequency logs for each tested configuration was conducted, Figure 9.

### 3.5. The Relevant Frequency Range

As a basic point for investigation of dynamical response, a frequency range needs to be determined. Taking into consideration Equations (7) and (8) and the relation between angular and ordinary frequency ω = 2πf, a following arises:

(9) $$f_{n}\;=\;n\frac{v}{2L},\;\;\mathrm{where}\;n\;=\;1,\;2,\;3\ldots \;$$

The wave velocity in a steel bar can be calculated from the ratio of the elasticity and the density of the steel. It is 5048 m/s. In this case, the first three vibration frequencies of the 2100 mm steel rod are 1202, 2404, and 3060 Hz, as shown in Table 1. Comparing these first three calculated frequencies with the measured values in the laboratory shows only slight differences (up to 2.70%). Furthermore, the average wave velocity of a 2000 m long grout (length of bar minus the threads) is 3824 m/s (determined by conducted ultrasound laboratory tests) so the first three frequencies, using Equation (9), have values of 956, 1912, and 2868 Hz.

In the case of the fully grouted model (fg_model), comprising a 2100 mm long bar and 2000 long grout section, both bar and grout have an influence on the natural frequencies of the model. Therefore, these elements can be considered as parallel coupled resistors which provide a resistance to the motion of the wave through the model. In this case, the wave velocity can be calculated as follows:

(10) $$\;v_{fg\_model}\;=\;\frac{v_{\mathrm{steel}}\cdot v_{\mathrm{grout}}}{v_{\mathrm{steel}}\;+\;v_{\mathrm{grout}}}$$

The wave velocity for fully grouted model is 2176 m/s. The first three frequencies are then 544, 1088, and 1632 Hz, as shown in Table 1. Given the many factors that are the result of the expected error of the experimental model relative to the above calculations, it can be concluded that the matching of the first three frequencies is relatively good and a relevant frequency range could be established. The fact that the presence of grout causes a decrease of the wave velocity in rock bolts was stated in the literature [24].

Further, calculation of natural frequencies of the partially grouted models (pg_models) can be analytically calculated by considering the grouted sections as the parallel coupled resistors which are connected in series with the non-grouted sections. A coefficient of grouting percentage (µ) has to be implemented into equation, which has a form:

(11) $$v_{pg\_model}\;=\;\left(\frac{v_{\mathrm{steel}}\cdot v_{\mathrm{grout}}}{v_{\mathrm{steel}}\;+\;v_{\mathrm{grout}}}\right)\cdot \mu \;+\;v_{\mathrm{steel}}\cdot \left(1\;-\;\mu \right)$$

The properties of a grout are implemented in Equation (11) through the velocity, based on Young’s elasticity modulus and density obtained from laboratory tests conducted on grout samples taken during construction of models. For example, in case of a 70% grouted rock bolt model (µ = 0.7), wave velocity is 3038 m/s, which gives the values of first three frequencies 723, 1447, and 2170 Hz. It is worth noting that Equation (11) determines the wave velocity based on grouting percentage and grout properties. The position of the defects in grouting are not covered by this equation.

### 3.6. Regression Analysis

**Figure 10:**
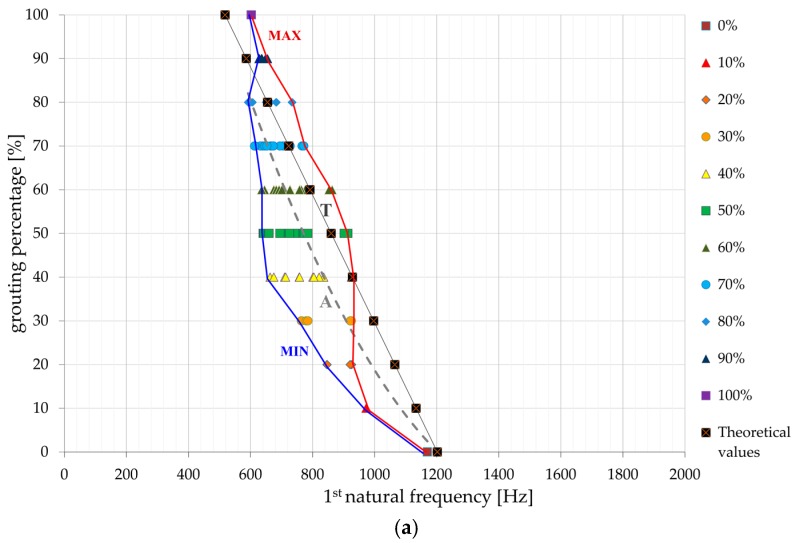

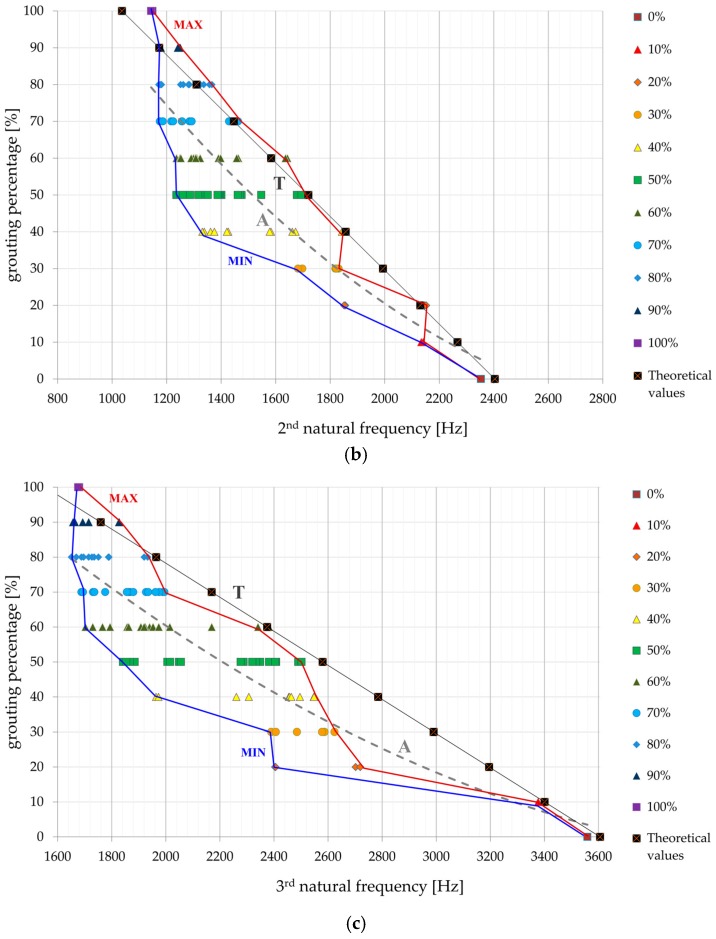
Regression functions for natural frequencies vs. grouting percentage for: (**a**) first, (**b**) second and (**c**) third natural frequency.

Figure 10 shows the first three frequencies of each testing combination, set in the corresponding diagrams. Here, the distribution scatter plots show the pairs of values of natural frequency vs. grouting percentage. The presented scatter plots are a starting point in the correlation and regression analysis for establishing a connection between the grouting percentage and the first three natural frequencies for the whole dataset for each frequency (average trendline marked as ‘A’). As an optimal regression function, the second order polynomial function was applied. The fitting uses the least squares method to minimize the squares of the residual deviations. The value of the coefficient of correlation implies no correlation or minor correlation when 0 ≤ R < 0.2, mild correlation for 0.2 ≤ R < 0.4, significant correlation for 0.4 ≤ R < 0.7, and high or very high correlation for 0.7 ≤ R ≤ 1.0. In this case, the correlation coefficients R of the regression polynomial functions describing the overall dependence of the natural frequencies on the grouting percentage have the values of 0.79 (first natural frequency), 0.83 (second natural frequency), and 0.86 (third natural frequency). These values of correlation coefficients clearly demonstrate benefits of analyzing first, second, and third natural frequency for determination of the grouting percentage along the rock bolt. Therefore, better understanding of rock bolt dynamic response was achieved when compared to the Kovačević et al. [3] study where it was shown that there is no clear correlation when only dominant frequency is observed. The diagrams on Figure 10 also show the theoretical function (T curves), obtained by the means of Equations (10) and (11). The trend and values of a T-curve from Figure 10, are influenced by the grouting percentage and grout characteristics. The overall trend of natural frequency increase with the decrease of grouting percentage is evident for both theoretical and experimental curves. However, certain deviations of experimental results are noticed in comparison to theoretical values and these can be attributed to additional influence of grout position on natural frequencies values. Additionally, minimum and maximum boundaries for each natural frequency are stressed out.

## 4. Numerical Modelling of Grouting Defects

In order to verify the experimental results and to further analyze the applicability for rock bolts installed in rock mass, a numerical modelling was conducted. Within the scope of this paper, two groups of rock bolt models were selected among the experimental models, as shown in Figure 5:

(A) Group I refers to the models that have a regular increase in the length of the grouted section.
(B) Group II refers to randomly selected rock bolts from each group of different grouting percentages.

### 4.1. Determination of Numerical Input Parameters

The laboratory test presented in Section 3.2 were conducted for all samples taken for the preparation of all the rock bolt models. An overview of material characteristics used as input for all numerical simulations is given in Table 2.

### 4.2. Composition of Numerical Models

Numerical analyses were carried out using a finite element method Ansys Workbench computer program, which is widely accepted in a wide range of human activities and is very often applied for scientific research purposes. Of the many modules offered by Ansys Workbench, in this case, the Harmonic Response module was used, since it enables the acquisition of a full frequency spectrum for the result of the force applied to the rock bolt head.

To carry out the analysis, the following steps were followed:

1. Design of geometric models.
2. Selection of a constituent model and bonds.
3. Input of the physical characteristics of the individual parts of the model.
4. Load input.
5. Discretization of models with appropriate elements.
6. Numerical calculation using the FEM method.
7. Overview and interpretation of results.

**Figure 11:**
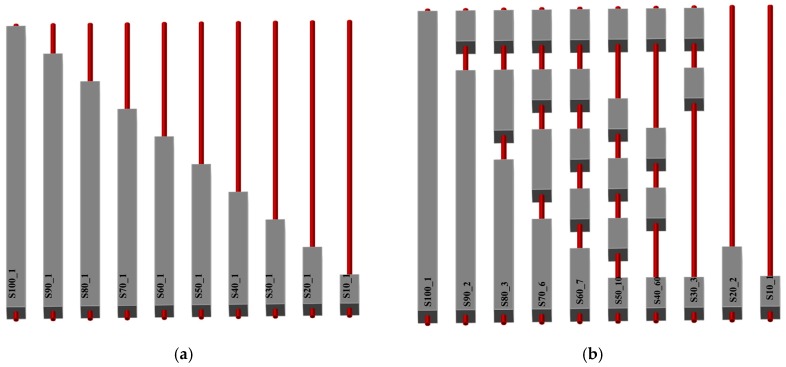
Rock bolt geometrical models: (**a**) Group I and (**b**) Group II.

Geometrical models were created using the Autocad 3D 2019 computer program, which is a sophisticated universal utility that supports three-dimensional modelling of complex objects. The advantage of its application is its simplicity and compatibility with the selected Ansys Workbench program. For the purposes of the analysis, the two groups of rock bolts that were analyzed through the experimental setup, Group I and Group II, were modelled. They are shown in Figure 11.

**Figure 12:**
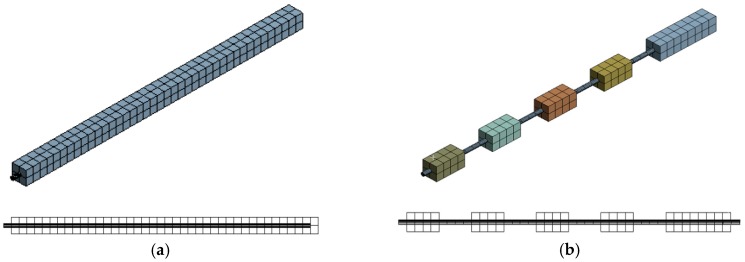
Discretization of numerical models: (**a**) S100_1 and (**b**) S60_7.

Since the numerical analyses in the presented research are dynamic analyses, which imply small strains, the constituent model chosen for all materials is linear-elastic, which assumes that the stresses are directly proportional to deformation. Once the constitutive model and the material characteristics of the material are defined, it is necessary to define the bonds between the individual materials. Since this numerical modelling employs a linear dynamic analysis, it is recommended to avoid nonlinear contacts. Therefore, the “no separation” or “bonded” contacts from a range of types of contact between the steel and the grout could be chosen. The “no separation” contact configuration can be applied to the sides of the region or to its edges, and by its application the separation of the sides that are in contact is not allowed, but a little slip of the sides may appear without friction. However, in case the so-called “bonded” type of contact is chosen, the 3D contact surface–surface element (contact 174) is selected for simulation of contacts between the target surfaces (steel–grout). A bonded contact, used in this research, can be applied to all regions that are in contact and in that case sliding or separation is not allowed, and the bodies tied to this contact act as mutually glued. As a method for simulating contacts, the so-called MPC (multi-point constraint) method is chosen where the contact is directly and efficiently formulated due to the internal addition of edge shift equations to match the displacement between the contact points. Since the magnitude of the force applied to the rock bolt head was not considered in experimental testing, it did not have a significant impact on the numerical modelling results. Namely, by varying the force magnitude, frequency spectra with equal values of natural frequencies were obtained which, depending on the magnitude of the force applied, have different amplitudes and these are of no interest for the subject of this research. Since the load magnitude has no effect on the value of the natural frequencies but only on their amplitudes, an anchorage load of 1 N has been imposed on the rock bolt head in all numerical analyses, in the direction of the anchor axis. The model discretization was performed in such a way that the steel bar was divided into 50 mm length elements while the grout was divided into 50 × 50 × 50 mm bodies. With mesh refinement, the numerical results are closer to the experimental, but the calculation time is prolonged. Figure 12a shows the discretization of the fully grouted model (S100_1), while Figure 12b shows the discretization of the rock bolt model S60_7 from Group II.

### 4.3. Comparison of Numerical Modelling and Experimental Results

**Figure 13:**
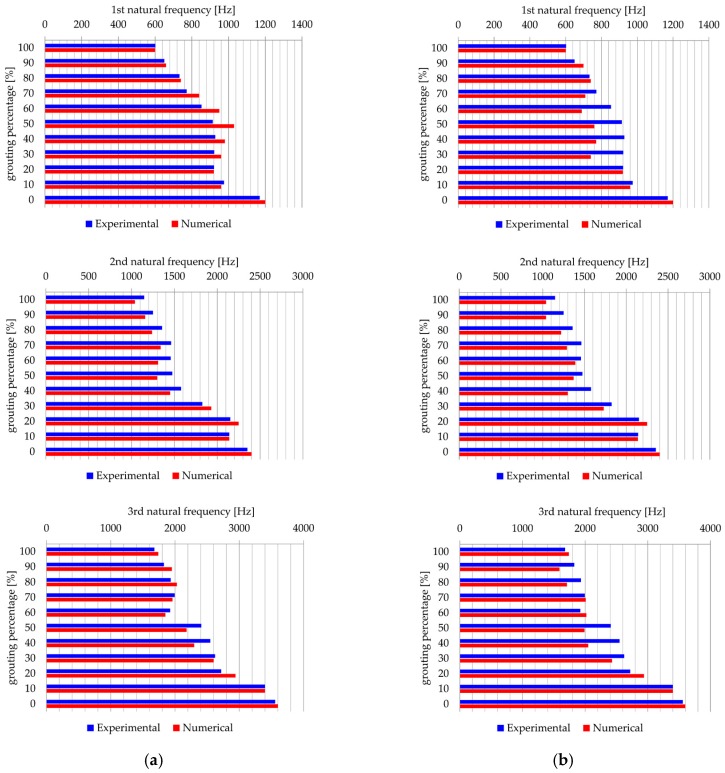
Comparison of the values of first, second, and third calculated natural frequency and experimentally obtained values for: (**a**) Group I and (**b**) Group II.

The results of the numerical analysis are presented as a comparison of the first three natural frequencies with the results of the laboratory model testing of models from Group I and Group II. Figure 13 shows the comparison between calculated and measured values of natural frequencies for the Group I and Group II, respectively.

The difference between the numerically calculated first three frequencies and the experimentally measured first three frequencies for both groups of selected groups of models, Group I and Group II, are up to 12%, which can be considered acceptable taking into account possible irregularities in the production of laboratory models (despite the strictly controlled conditions), as well as variations in the measuring environment. Accepting the above, the further numerical calculations include the consideration of grout stiffness variation influence on the dynamic response of models.

### 4.4. Analysis of Grout Stiffness Influence

Following the comparison of numerical and experimental results, the next phase of numerical modelling implies variation of small strain stiffness of grouts for all models within Groups I and II, in order to evaluate the influence of stiffness on the natural frequencies. Considering that the numerical input, besides values of elasticity modulus, requires a density value input, it is selected in all analysis as constant reference value of 2000 kg/m^3^. Used values of elasticity modulus, density, and (calculated) wave velocities are shown in Table 3.

**Figure 14:**
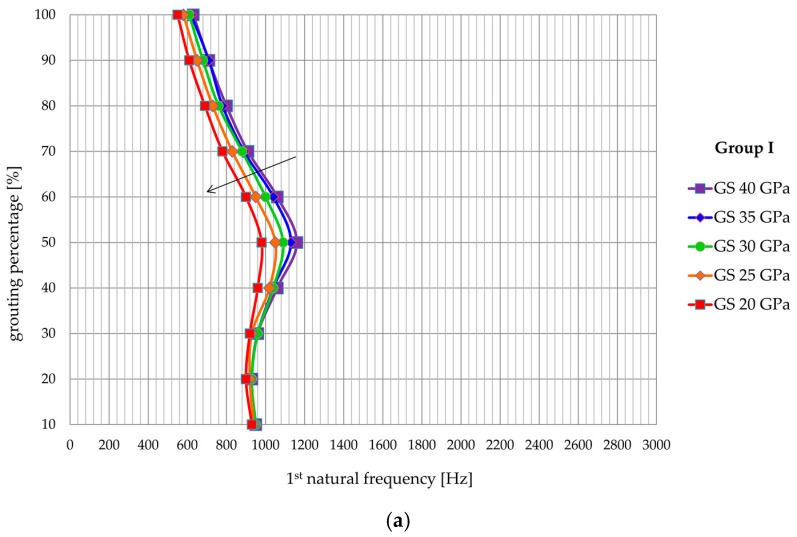

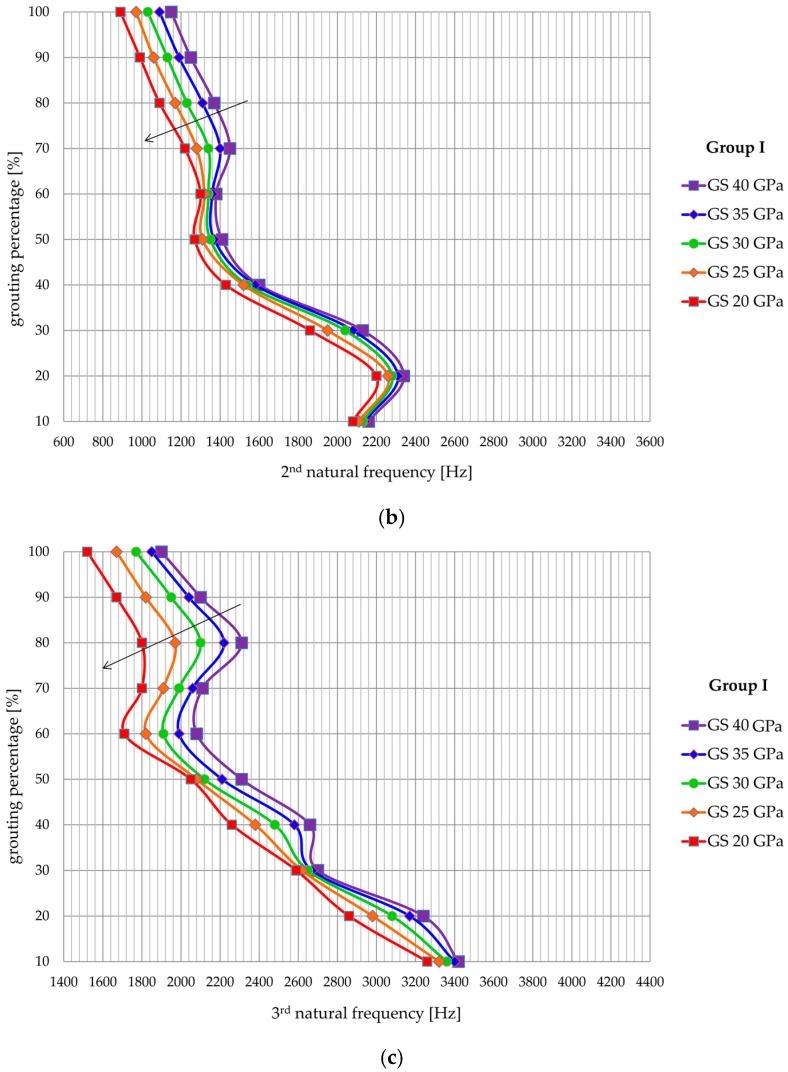
Influence of grout stiffness on (**a**) first, (**b**) second, and (**c**) third natural frequency of Group I models.

**Figure 15:**
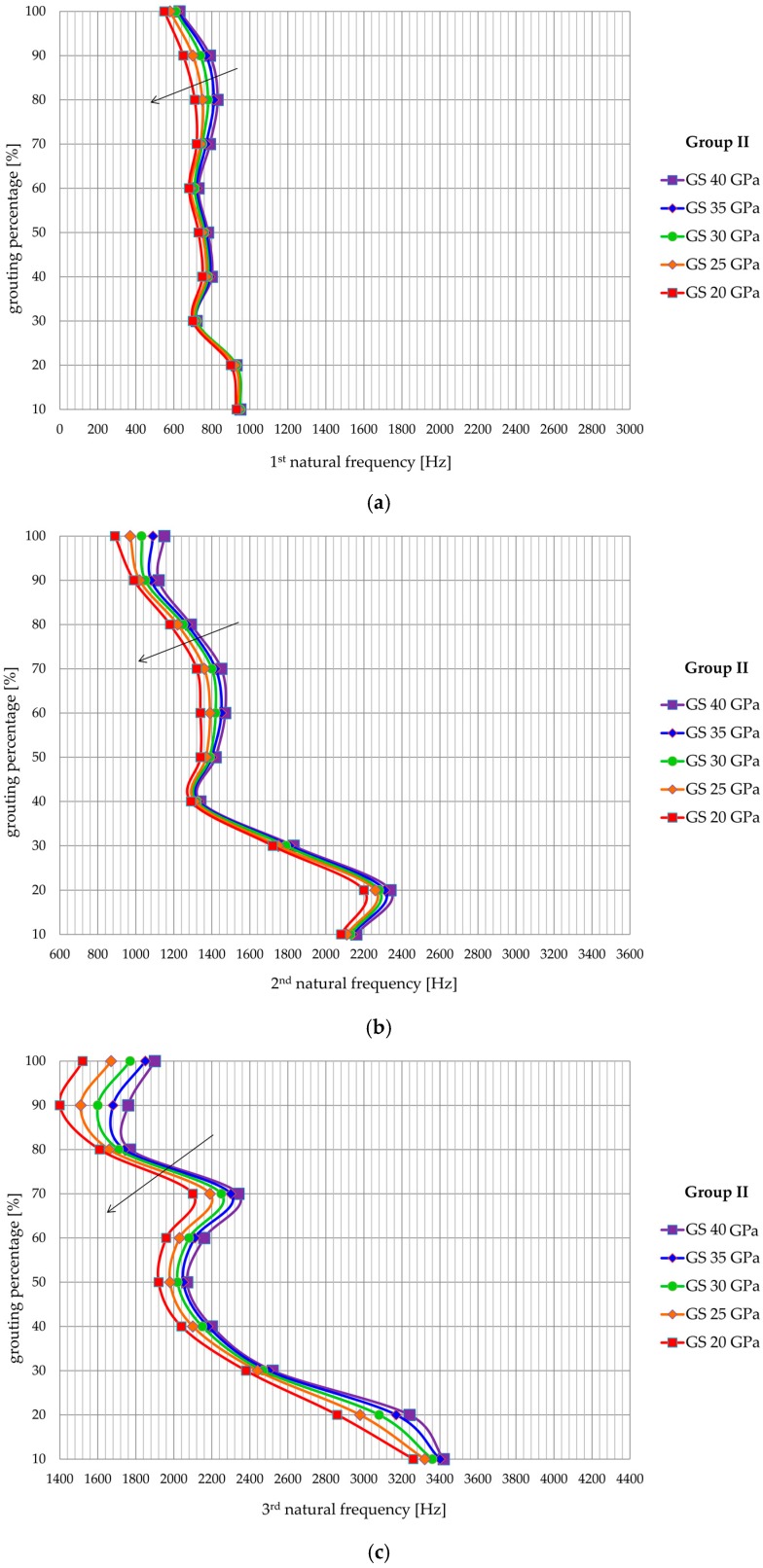
Influence of grout stiffness on (**a**) first, (**b**) second, and (**c**) third natural frequency of Group II models.

A range of elasticity modulus from 20 to 40 GPa is varied since these values represent commonly achieved values for rock bolt systems. Figure 14 shows the influence of the modulus of elasticity of the grouting mixture on the values of the natural frequencies for Group I, while in Figure 15 shows the same for Group II. A clear reduction of the natural frequency values is apparent from Figure 14 and Figure 15 with a decrease of the modulus of elasticity of the grout. This reduction is least pronounced for the first natural frequency and is most clearly expressed for the third natural frequency. In addition, the reduction of the value of the natural frequencies is generally more significant in the models with a higher percentage of grouting, although locally there are values that do not meet the specified. The variation of the elastic modulus does not, however, affect the general trend of increasing natural frequency with the reduction in grouting percentage, whereby the shape of the natural frequency—the grouting percentage curve is independent of the value of modulus of elasticity.

## 5. Conclusions

An experimental study including determination of a correlation between grouting percentage and first three natural frequencies of a laboratory rock models was conducted. In total, 94 different combinations of different grouting percentages and positions were tested. Prior to testing, a data acquisition setup was established consisting of a custom-made nut for positioning of the accelerometer, a hammer made of soft steel for the generation of a dynamical impulse, a vibration input module, and a developed software program. By analyzing the statistical distribution of the data and by establishing a regression function (second order polynomial functions were employed) and regression coefficients, it was shown that a high correlation exists between the natural frequencies and the grouting percentage. This clearly demonstrates the benefits of analyzing first, second, and third natural frequency for determination of grouting percentage along the rock bolt and provides better understanding of rock bolt dynamic response when compared to previous studies which analyzed only dominant frequency. Following experimental testing, the objectives of the numerical simulation included comparison with experimental results, as well as the analysis of the effect of the grout stiffness on the frequency response of rock bolts. When comparing the results of numerical analyses with the results of experiments, a similar general increasing trend of the first three natural frequencies with a reduction in grouting percentage is clearly visible. Moreover, the values of the natural frequencies obtained by numerical analysis have approximately the same values as the natural frequencies obtained by experimental testing. Numerical analyses have also shown that the reduction of the modulus of elasticity of the grouting mixture, leads to the reduction of the values of the natural frequencies while the variation of the elastic modulus does not affect the general trend of increasing natural frequencies with the reduction in grouting percentage, whereby the shape of the natural frequency—the grouting percentage curve is independent of the modulus of elasticity. The potential of using numerical methods, which are properly calibrated, for analysis of more complex grouting defects is therefore evident. Overall findings of this study are in establishing a clear correlation between first, second, and third natural frequency of rock bolt and the percentage of grouting along the rock bolt. After the proposed grouting evaluation method is validated in situ condition, it can be used as a control quality measure to check whether the installation procedures conform with the design requirements. However, because of the change of natural frequencies with rock bolt load increase, the method is applicable only to non-loaded rock bolts, tested immediately after their installation.

## Figures and Tables

**Table 1:** Comparison of calculated and measured first three natural frequencies of steel bar and fully grouted model.

	**Steel Bar (No Grout)**		
	**Calculated [Hz]**	**Measured [Hz]**	**Difference [%]**
f_1_	1202	1170	2.70
f_2_	2404	2353	2.10
f_3_	3606	3559	1.30
	**Fully Grouted Model**		
	**Calculated [Hz]**	**Measured [Hz]**	**Difference [%]**
f_1_	544	603	9.78
f_2_	1088	1143	4.81
f_3_	1632	1676	2.63

**Table 2:** Material properties of steel bar and grout mixtures.

**Steel Bar, B500B**	
Elasticity Modulus [Pa]	2 × 10^11^
Density [kg/m^3^]	7850
Poisson’s Coef. [—]	0.30
**Grouting Mixture, w/c = 0.42**	
Elasticity Modulus [Pa]	2.2 × 10^10^ to 2.7 × 10^10^
Density [kg/m^3^]	1800 to 2000
Poisson’s Coef. [—]	0.18

**Table 3:** Parameters for analysis of grout stiffness influence.

Analysis No.	Elasticity Modulus [Pa]	Density [kg/m^3^]	Wave Velocity [m/s]
1	4.0 × 10^10^	2000	4472
2	3.5 × 10^10^	2000	4183
3	3.0 × 10^10^	2000	3873
4	2.5 × 10^10^	2000	3536
5	2.0 × 10^10^	2000	3162
